# Supplementary material for: Change in the Gut Microbiome and Immunity by Lacticaseibacillus rhamnosus Probio-M9
Source: Microbiol Spectr. 2023 Mar 13;11(2):e03609-22. doi: 10.1128/spectrum.03609-22 (PMC10100958; doi:10.1128/spectrum.03609-22)

Supplementary Materials for

The change of the gut microbiome and immunity by *Lacticaseibacillus rhamnosus* Probio-M9

The additional tables include:

Table S1. The immune function test results of all subjects at the beginning and end of the intervention.

Table S2. The profiling results for all microbes from the probiotic and placebo group at the species level.

Table S3. The SNVs in the two groups.

The supplementary figures include:

Figure S1. Comparison of the relative abundance of *Lacticaseibacillus rhamnosus* between the two groups at different time points.

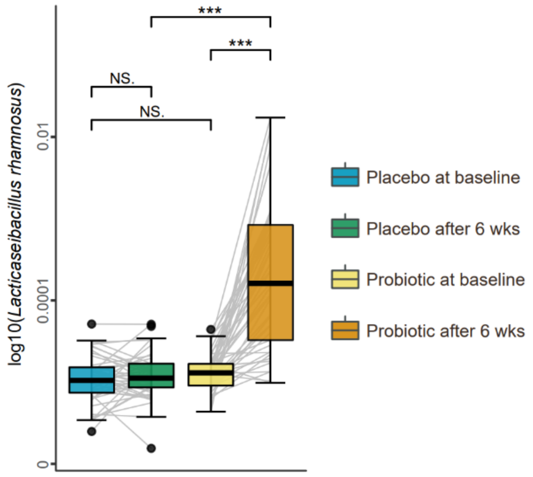

Supplement: Supplemental file 1 — Supplemental material. Download spectrum.03609-22-s0001.pdf, PDF file, 0.2 MB [file spectrum.03609-22-s0001.pdf]
